# Concentration and Methylation of Cell-Free DNA from Blood Plasma as Diagnostic Markers of Renal Cancer

**DOI:** 10.1155/2016/3693096

**Published:** 2016-09-20

**Authors:** Inessa Skrypkina, Liudmyla Tsyba, Kateryna Onyshchenko, Dmytro Morderer, Olena Kashparova, Oleksii Nikolaienko, Grigory Panasenko, Sergii Vozianov, Alina Romanenko, Alla Rynditch

**Affiliations:** ^1^Department of Functional Genomics, Institute of Molecular Biology and Genetics of the National Academy of Science of Ukraine, Kyiv, Ukraine; ^2^Department of Molecular Oncogenetics, Institute of Molecular Biology and Genetics of the National Academy of Science of Ukraine, Kyiv, Ukraine; ^3^Institute of Urology, National Academy of Medical Sciences of Ukraine, Kyiv, Ukraine

## Abstract

The critical point for successful treatment of cancer is diagnosis at early stages of tumor development. Cancer cell-specific methylated DNA has been found in the blood of cancer patients, indicating that cell-free DNA (cfDNA) circulating in the blood is a convenient tumor-associated DNA marker. Therefore methylated cfDNA can be used as a minimally invasive diagnostic marker. We analysed the concentration of plasma cfDNA and methylation of six tumor suppressor genes in samples of 27 patients with renal cancer and 15 healthy donors as controls. The cfDNA concentrations in samples from cancer patients and healthy donors was measured using two different methods, the SYBR Green I fluorescence test and quantitative real-time PCR. Both methods revealed a statistically significant increase of cfDNA concentrations in cancer patients. Hypermethylation on cfDNA was detected for the* LRRC3B* (74.1%),* APC* (51.9%),* FHIT* (55.6%), and* RASSF1* (62.9%) genes in patients with renal cancer. Promoter methylation of* VHL* and* ITGA9* genes was not found on cfDNA. Our results confirmed that the cfDNA level and methylation of CpG islands of* RASSF1A*,* FHIT*, and* APC* genes in blood plasma can be used as noninvasive diagnostic markers of cancer.

## 1. Introduction

Renal cell carcinoma (RCC) is a widespread oncologic disease that accounts for about 3% of all malignancies in adults and 85% of all primarily malignant tumors in kidney [1]. Metastases detected at the time of establishing a diagnosis are present in 25–30% of patients, and even after surgery the disease progresses in 20–30% of patients [2, 3]. An asymptomatic period of the disease makes early diagnosis of this type of tumor difficult to perform. Globally, the incidence rates of kidney cancer are predicted to increase. The International Agency for Research on Cancer claims that this number will rise to 22%, from 337,860 cases in 2012 to 412,929 cases in 2020 [4].

Clear cell carcinoma is the most common type of RCC, accounting for 70–80% of all RCCs [5]. Development of this particular type of RCC is associated with many tumor suppressor genes that are localized in the short arm of human chromosome 3. They can be inactivated as a result of mutations, LOH (loss of heterozygosity), or methylation of CpG islands in promoter regions [6–9]. Identification of aberrantly methylated genes for a particular tumor type can be helpful in early diagnosis of the disease.

Cell-free DNA (cfDNA) enters the blood stream from apoptotic and necrotic tumor cells and is useful in detecting tumor-specific signatures, including the methylation of genes [10, 11]. Aberrant cfDNA methylation has been described in most cancer types and is being actively investigated for minimally invasive clinical diagnostics [11–13].

Large-scale NotI-microarray analyses of genetic and epigenetic alterations in the genes of chromosome 3 in RCC revealed that leucine-rich repeats containing 3B (*LRR*C3B) and Von Hippel-Lindau (*VHL*) genes possess the highest frequency of deletions and/or methylations in renal carcinoma [14, 15]. Adenomatosis-polyposis-coli (*APC*), Ras association domain family 1 (*RASSF1*), and fragile histidine triad (*FHIT*) genes were shown to have high levels of methylation in cfDNA and/or in renal tumors [16–22].

In this study we determined the plasma cfDNA concentration (by quantitative PCR and the fluorescence test) and analysed methylation of 6 genes (*APC*,* FHIT*,* RASSF1*,* LRRC3B*,* VHL*, and* ITGA9* (Integrin *α*9*β*1)) in plasma samples from patients with kidney cancer in order to evaluate the diagnostic value of these markers for cancer detection.

## 2. Materials and Methods

### 2.1. Sample Collection

The study included 27 patients undergoing surgery for kidney cancer at the Institute of Urology, National Academy of Medical Sciences of Ukraine in Kyiv, between January 2011 and August 2011. Before surgery all patients were fully examined according to the protocols of the Ministry of Health of Ukraine: laboratory clinical analysis, Doppler ultrasound diagnosis, renal scintigraphy, and spiral computed/magnetic resonance tomography of the retroperitoneal space. For the negative controls, peripheral blood was collected from 15 healthy individuals. All patients gave written informed consent prior to enrollment in the study. The samples were collected in accordance with the Declaration of Helsinki and the guidelines issued by the Ethics Committee of the Institute of Urology NAMS of Ukraine. The Ethics Committee of the Institute of Urology specifically approved this study.

### 2.2. Extraction of сfDNA

Blood (5 mL) was collected in K3 EDTA-containing tubes (Cat. number 2102, APTACA, Italy). The samples were stored at 4°C and treated within 3 h after blood collection. The plasma was isolated by low-speed centrifugation: 250 ×g for 7 min, 350 ×g for 8 min, and 500 ×g for 10 min using Jouan MR23i centrifuge (JOUAN, France). It was then aliquoted and cryopreserved at −70°C.

cfDNA was isolated from 2 mL plasma using the Proba NA Kit (DNA-Technology, Russia) according to the manufacturer's recommendations (final elution volume was 150 *μ*L). The extracted DNA was subjected to PCR with the* ACTB* gene (5′-CCACACTGTGCCCATCTACG-3′ and 5′-AGGATCTTCATGAGGTAGTCAGTCAG-3′; 99 bp fragment) as control, and the PCR products were examined by electrophoresis (see Supplementary Figure S1 in Supplementary Material available online at http://dx.doi.org/10.1155/2016/3693096). PCR conditions were as follows: 95°C for 4 min and then 40 cycles of 95°C for 40 s, 56°C for 20 s, and 72°C for 30 s, with a final extension for 5 min at 72°C.

### 2.3. Quantification of Plasma cfDNA by Real-Time PCR

To measure the plasma cfDNA concentration, the genomic sequence of *β*-actin was amplified by quantitative real-time PCR (qPCR). The primers and fluorescent probe used for qPCR were as described in Herrera et al. [23]. 5 *μ*L purified cfDNA was amplified using 0.3 *μ*M of each primer (5′-CCACACTGTGCCCATCTACG-3′ and 5′-AGGATCTTCATGAGGTAGTCAGTCAG-3′) and a 0.25 *μ*M fluorescent probe (5′-FAM-ATGCCCTCCCCCATGCCATCCTGCGT-TAMRA-3′). The length of the amplified fragment was 99 bp. PCR was performed under the following conditions: 10 min at 95°C, followed by 40 cycles of 15 s at 95°C and 1 min at 60°C. Quantitative standard curves were prepared using serial dilutions (from 20 pg to 100 ng/reaction) of control genomic DNA. Human HCT116 DKO Nonmethylated DNA (Cat. number D5014-1, Zymo Research Corporation, USA) was used as calibrator for quantification. The concentration of control DNA was assessed using a NanoDrop 2000 spectrophotometer (Thermo Scientific, USA). No-template controls (NTCs) were used as negative controls. The fluorescence of the amplified PCR products was detected using the BioRad iQ5 Optical System (Bio-Rad, USA). The results of the qPCR assays represent the mean of three independent experiments, each consisting of duplicate samples. The analysis was repeated if the difference between duplicate samples was greater than one cycle threshold. The linear dynamic range was determined by the standard curve and correlation coefficients (*R* ^2^), which was ≥0.98. A more detailed version of the protocol is given in Supplementary Table S1. The statistical significance of differences between samples was established using the Mann-Whitney *U* test.

### 2.4. Quantification of Total Plasma DNA by the Fluorescence Test

Evaluation of the cfDNA concentration was also performed measuring the fluorescence of intercalating dye [24]. Specifically, 5 *μ*L of a sample or the same volume of a standard dilution of genomic DNA (Human HCT116 DKO Nonmethylated DNA) with known concentration (0 ng/mL and 9 serial dilutions from 1 to 256 ng/mL) was added to 195 *μ*L of a SYBR Green I solution (Cat. number S7585, Thermo Fisher Scientific, USA) in PBS buffer (1 : 10,000) and to black 96-well plates (PAA, Cat. number PAA30296X, Austria) and incubated for 10 min. Two to three identical mixtures were prepared from each sample or standard for greater accuracy. The fluorescence of the mixtures obtained was measured by the “VICTOR^3^ 1420-050” Multilabel Plate Readers (Perkin Elmer, USA) using filters for FITC (485/535 nm) and 1 s acquisition time. The DNA concentration was calculated from the standard curve (*R* ^2^ was 0.97).

### 2.5. Evaluation of Gene Methylation Status

Bisulfite treatment of isolated DNA was performed using the EZ DNA Methylation Kit (Cat. number D5001, Zymo Research Corporation, USA) according to the manufacturer's instructions. The methylation status of the different genes was determined qualitatively by the methylation-specific polymerase chain reaction (MS-PCR) [25]. Real-time MS-PCR was performed in a Bio-Rad iQ5 Real-Time PCR detection System (Bio-Rad, USA). Primer sequences used for MS-PCR analysis, with PCR product size and primer annealing temperature, are listed:* RASSF1* methylated-specific forward, 5′-GTGTTAACGCGTTGCGTATC-3′ and reverse, 5′-AACCCCGCGAACTAAAAACGA-3′ (60°C, 93 bp) [26];* FHIT*, 5′-TTGGGGCGCGGGTTTGGGTTTTTACGC-3′ and 5′-CGTAAACGACGCCGACCCCACTA-3′ (62°C, 74 bp) [27];* APC*, 5′-TATTGCGGAGTGCGGGTC-3′ and 5′-TCGACGAACTCCCGACGA-3′ (60°C, 98 bp) [28];* LRRC3B*, 5′-GGTGCGAGGAAGGTAGGC-3′ and 5′-ACCAATACCTCGCCGACG-3′ (64°C, 149 bp) [29];* VHL*, 5′-TGGAGGATTTTTTTGCGTACGC-3′ and 5′-GAACCGAACGCCGCGAA-3′ (60°C, 158 bp) [30];* ITGA9,* 5′-TGGAGTATTTTTACGATAATACGC-3′ and 5′-AAAAACCGAAAAAACGACGA-3′ (64°C, 116 bp) [31]. Two *μ*L of bisulfite-modified DNA was subjected to PCR amplification in a final reaction volume of 25 *μ*L 1x Maxima SYBR Green qPCR Master Mix (Cat. number K0251, Thermo Scientific, USA) and 0.3 *μ*M of each primer. PCR was performed with an initial 10 min incubation at 95°C, followed by 45 cycles of denaturation at 95°C for 15 s, annealing for 20 s, extension at 72°C for 30 s, and a final 7 min hold at 72°C. Each sample was assayed in triplicate, and each run included water blanks and an external control (universal methylated DNA). A fully methylated positive control was created by treating the DNA of lymphocytes from healthy donors with* SssI* CpG Methyltransferase (Cat. number EM0821, Thermo Scientific, USA) according to the manufacturer's recommendations. The specificity of the PCR products was confirmed by melting curve analysis. To verify MS-PCR data, the MSP sequencing assay was performed using Genetic Analyser 3130 (Applied Biosystems, USA) following manufacturer's protocols.

### 2.6. Statistical Analysis

Samples sizes were calculated using the formula described in [32] assuming *α* and *β* values of 0.05 and 0.2, respectively. We used standard deviation obtained in our preliminary experiments and estimated 150% difference in means.

To evaluate the statistical significance of differences between groups we performed the nonparametric Mann-Whitney *U* test using the OriginPro 9.1 software (OriginLab, USA) or the Chi-square test (*χ* ^2^) using Microsoft Excel 2007 in the case of categorical variables.

Differences were considered statistically significant if *p* < 0.05. To evaluate the discriminative power of the parameters studied for kidney cancer diagnostics we built binary logistic regression models for the selected predicting variables and all their possible combinations using SPSS version 22 (IBM, USA). From these models, the probabilities of positive outcome (i.e., cancer occurrence) were calculated. These probabilities were used for Receiver-operating characteristics (ROC) analysis. Building of ROC and evaluation of AUC (Area Under Curve) was performed using the GraphPad Prism 6.07 (GraphPad Software, La Jolla, CA, USA) or the OriginPro 9.1 software (OriginLab, USA).

## 3. Results

### 3.1. Concentration of cfDNA in Blood Plasma of Patients with Renal Cancer and of Healthy Donors

In this study, blood samples from 27 patients with renal cancer and from 15 healthy donors were used. The blood samples were collected before surgery in the Institute of Urology NAMS of Ukraine. The results of the histological examination of tumors showed that 23 patients had clear cell carcinoma, 2 patients had sarcoma-like tumors, 1 patient had mixed type RCC (papillary/clear cell), and 1 patient had cancer of the renal pelvis (Table 1).

The concentrations of cfDNA in blood plasma were determined by two methods: by measuring the fluorescence level of intercalated SYBR Green I dye and by quantitative real-time PCR (qPCR) of the *β*-actin gene.

**Figure 1:**
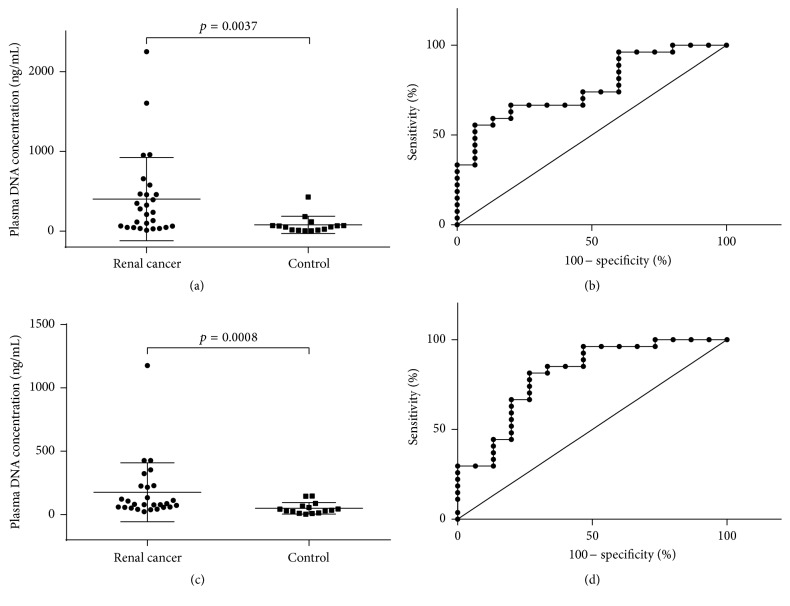
Analysis of cfDNA concentration in plasma of patients with renal carcinoma and controls. cfDNA concentrations were determined by measuring the fluorescence level of intercalated SYBR Green I dye (a) and by qPCR (c). ROC curve analysis of cfDNA concentration in cancer patients compared with the control group ((b) fluorescence test; (d) qPCR).

The results of the SYBR Green I fluorescence measurements showed that the concentrations of cfDNA in patients with renal cancer range from 11.3 to 2249.12 ng/mL (median 235.55 ng/mL). The range of cfDNA concentration in healthy donors was much lower, from 3.29 to 426.75 ng/mL (median 53.66 ng/mL) (Figure 1(a)).

qPCR revealed a statistically significant increase of cfDNA concentration in cancer patients (median 80.97 ng/mL, range 23.3–1176.6 ng/mL of plasma). As can be seen from the box plot (Figure 1(c)), these values are significantly higher in RCC patients compared to healthy donors (median 35.1 ng/mL, 3.0–146.78 ng/mL of plasma) (Figure 1(c)).

Receiver-operating characteristics (ROC) analysis showed that the concentration of cfDNA can be used as diagnostic feature for the detection of renal tumors (Figures 1(b)–1(d)). AUC obtained for qRCR analysis was slightly higher (0.8049, *p* = 0.0012) than for the SYBR Green I fluorescence measurements (0.7679, *p* = 0.0044) (Table 2).

### 3.2. Analysis of Methylation of Tumor Suppressor Genes in cfDNA

Since the cfDNA level alone cannot be a specific marker of renal cancer [11], we also analysed the methylation status of CpG islands of 6 tumor suppressor genes in the cfDNA. Using bisulfite treatment followed by MS-PCR we detected methylation of the* LRRC3B, APC, FHIT, *and* RASSF1* genes in the cfDNA of cancer patients. Promoter methylation of the* LRRC3B* gene was detected in 20 out of 27 samples (74%); methylation of the* RASSF1, APC*, and* FHIT *genes was found in 17 (63%), 14 (52%), and 15 (55.6%) patients, respectively (see Table 3 for detailed methylation frequencies). Methylation was not detected in the* VHL *and Integrin *α*9*β*1 (*ITGA9*) genes in plasma cfDNA.

Analysis of simultaneous methylation of CpG islands of the* LRRC3B, FHIT, АPС, *and* RASSF1 *genes showed that all the samples from cancer patients contained at least one methylated promoter; two promoters were methylated in 33.3%, three promoters were methylated in 27%, and four methylated promoters were detected in 11.1% of the samples (Tables 3 and 4).

However,* LRRC3B* showed a low specificity as a marker of cancer, since it was methylated in 5 out of 15 (33.3%) healthy donors. Methylation of* FHIT* was not detected in the cfDNA of the control group, while methylation of the* APC* and* RASSF1 *genes was found in 1 out of 15 (6.7%) healthy donors. Methylation of* APC* and* RASSF1 *was detected in different healthy individuals (Table 4).

The sensitivity of each of these markers exceeded 50% and was 51.9% for* APC*, 63% for* RASSF1*, and 55.6% for* FHIT*, which exhibited the best specificity in our test (100%).

The use of the combined analysis of methylation status of three genes (*RASSF1*,* FHIT*, and* APC*) increased the sensitivity (77.8–92.3%), while the specificity remained high (86.7–93.3%) (Table 4). We did not find any correlation between hypermethylation, cfDNA concentration, and clinicopathological parameters (grade, lymph node metastasis, age, and sex) in patients with renal cancer.

**Figure 2:**
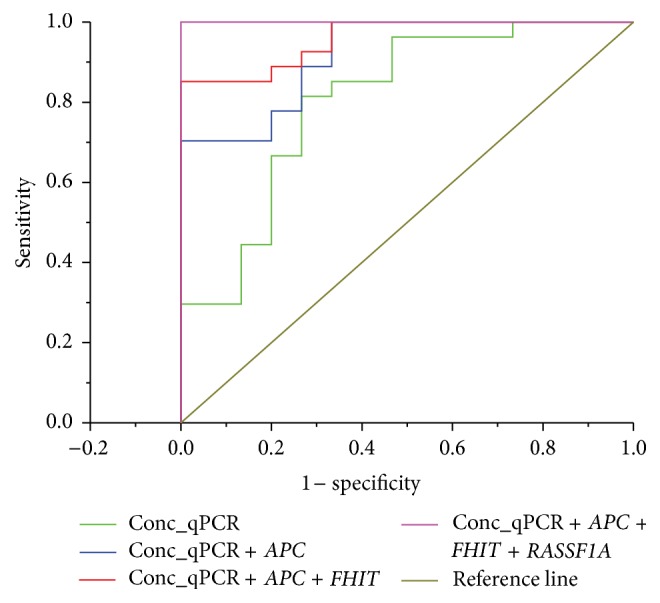
Receiver-operating characteristics (ROC) curves obtained by using different models for the discrimination between healthy controls (*n* = 15) and renal carcinoma patients (*n* = 27). Conc_qPCR: concentration of cfDNA determined by qPCR.

To explore the potential of combined cfDNA concentration and gene methylation for RCC diagnostics, we performed binary logistic regression modelling. As a predictor of variables we used cfDNA concentrations measured by quantitative PCR and methylation of* APC*,* FHIT*, and* RASSF1* genes. We built separate models for cfDNA concentration alone and for all possible combinations of cfDNA concentration and methylation of one, two, or three genes. Predictive properties of the models were compared by ROC analysis. As reported above, the AUC value for the cfDNA concentration alone was 0.8. Addition of one of the genes slightly increased the AUC to values 0.88–0.918, although these differences were not statistically significant as can be seen from 95% confidence intervals (Table 5). Addition of two genes led to further increase of the AUC value up to 1 when using* APC* and* RASSF1*. Finally, the AUC value was 1 when we used the cfDNA concentration and methylation of all three genes studied. The results of ROC analysis are summarised in Table 5; some of representative ROC curves are shown in Figure 2.

## 4. Discussion

The level of cfDNA in blood plasma could be a universal marker of malignancy [33]. Many studies have shown that changes in cfDNA concentration can be correlated with development, prognosis, and survival of cancer patients. An increase of cfDNA concentration was observed in patients with breast, gastric, ovary, lung, colon, and prostate cancer [11, 34–39]. It was suggested that an increase of cfDNA concentration in cancer patients is associated with apoptosis and necrosis of cancer cells in the tumor microenvironment [40]. This suggestion was supported by numerous cancer-specific alterations (such as allelic imbalances, methylation, and mutations) that were found in blood cfDNA (for reviews see [11, 41]). It was also demonstrated that monitoring of the cfDNA level in peripheral blood can be used as biomarker of response to therapy in different cancer types [38, 42, 43].

Previous studies demonstrated that the evaluation of concentration of low molecular weight cfDNA (up to 100 bp) is the most representative for detection of malignancies and disease prognosis since the level of fragments of this size increases with disease progression [44–47]. Recently, Lu et al. [47] showed that cfDNA fragments of 67 bp and 180 bp did not differ between the controls and nonmetastatic RCC patients, while the cfDNA integrity index decreased from control to the metastatic group. Significantly higher concentrations of low molecular weight fragments were found in the RCC patients [47]. Here we have shown an increase of cfDNA concentration in RCC patients using genomic cfDNA fragment of *β*-actin gene of 99 bp. Recent experiments from other laboratories also demonstrated increased cfDNA levels in the blood of patients with renal cancer compared to healthy individuals [19, 48, 49].

Absolute values of cfDNA concentrations obtained by two distinct methods are different (11–2249 ng/mL of plasma in the fluorescence test compared to 23–1177 ng/mL in qPCR), but in both cases they were significantly higher than in healthy individuals (4–426 ng/mL in the fluorescence test and 3–146 ng/mL in qPCR). The obtained results agree with data from other studies in which the determination of the cfDNA concentration using fluorescent dyes gave higher values than qPCR [24, 41]. The high AUC values obtained for both methods of cfDNA concentration measurement in RCC patients (0.7679 for fluorescent test and 0.80494 for qPCR) demonstrate that these methods can be used for clinical investigations. In addition to quantitative changes, cfDNA also possesses qualitative changes that occur in DNA of tumor cells, such as mutations, microsatellite instability, and methylation [11, 41, 50]. Methylation of gene promoters is a well-known mechanism of regulation of gene expression [51]. Most frequently, aberrant methylation of genes occurs in cancer cells. Aberrant methylation of the promoter detected in cfDNA can be used for noninvasive detection of cancer, differential diagnosis, prognosis of survival, and response to cancer therapy [52–57]. Currently, several diagnostic systems based on the detection of DNA methylation exist that are aimed not only at detecting malignancy (MethylMeter from RiboMed, USA), but also at detecting specific types of cancer (Epi proColon and Epi proLung from Epigenomics AG, Germany; Confirm MDx for Prostate Cancer from MDxHealth, USA; Product series DecisionDx G-CIMP, Melanom, EC, UM, Thymoma from Castle Biosciences). The search for new tools is being pursued because only two of these systems (MethylMeter and Epi proColon) can detect cancer at early stages of development and can monitor treatment since they are based on the detection of cfDNA methylation.

In this study we started investigating methylation of previously identified tumor suppressor genes in cfDNA. Data from many studies show that the* RASSF1* gene plays an important role in cancerogenesis. Hypermethylation of* RASSF1* CpG islands is associated with different types of cancer and with the risk of progression of tumorigenesis [58–64]. It was also shown that rat* RASSF1* is involved in early tumorigenesis of RCC [16, 17]. Studies on methylation of this gene in blood serum led to controversial results. Hauser et al. [18] showed that* RASSF1A *is methylated in 22.9% of patients; the study of De Martino et al. [19] demonstrated methylation of* RASSF1A* in 45.9% of patients; Hoque et al. [20] observed methylation of this gene in 11% of serum samples of patients with RCC. In our study methylation of* RASSF1 *was detected in 62.9% of patients. The differences in methylation levels of the* RASSF1* gene can be explained by the use of different CpG islands for analyses. We studied methylation of CpG region located within the first exon of* RASSF1C*, while Hauser et al. [18] and De Martino et al. [19] analysed the region located upstream of the initiation codon. Previously, it was reported that these two CpG islands were differentially methylated in melanoma cell lines and melanoma tumors [65]. Ellinger et al. [66] demonstrated a 100% correlation between DNA hypermethylation of the* RASSF1A* promoter and papillary RCC. However, De Martino et al. [19] analysed 31 samples of papillary RCCs and found no association of* RASSF1A* methylation with the histological subtypes of RCC. In our study* RASSF1* was also methylated in papillary RCC, but it was the only sample of this cancer subtype analysed.

Previously we reported changes in the* LRRC3B* gene promoter during the search for genetic and epigenetic alterations in chromosome 3 in epithelial tumors using NotI-microarrays [14, 67, 68].* LRRC3B* was identified by Kim et al. [69] as a putative gene suppressor of several tumors that are silenced in gastric cancers by epigenetic mechanisms. Increased methylation of the* LRRC3B* gene promoter was confirmed in samples of clear cell RCC and colorectal, head, and neck cancer [29, 70, 71]. A high level of* LRRC3B* hypermethylation was noted not only in RCC patients (74%), but also in healthy donors (33%) in our study, questioning the use of this gene for the diagnosis of renal cancer on cfDNA.

The promoter of the* APC* gene was methylated in 51.9% of patients, which is in good agreement with the results of Hauser and colleagues [18], who detected methylation of the* APC* gene in 54.3% of patients using cfDNA.

Previously, a significant correlation between* FHIT* expression in clear cell renal carcinomas and patient survival was demonstrated [21]. Kvasha et al. [22] showed a correlation between hypermethylation of the* FHIT* CpG island and a significant decrease of* FHIT* expression in clear cell RCC. The level of aberrant methylation of* FHIT*, obtained in our study on cfDNA (55.6%), was close to the results obtained in the study of Kvasha et al. in samples of RCC tumors (54.6%).

Integrin *α*9*β*1 plays an important role in various signal transduction pathways that control proliferation, migration, and differentiation of both normal (reviewed in [72, 73]) and cancer cells (reviewed in [74, 75]). Downregulation of* ITGA9* expression was observed in several cancer types [76–78] that could be caused either by mutations in this gene [79] or by hypermethylation [31, 68, 80]. However, methylation of the* ITGA9* gene was not detected in our experiments. We also have not identified methylation of the* VHL* gene, although NotI-microarray hybridization revealed high levels of changes in this gene (47%) in renal cancer [14]. It is possible that these changes are associated with deletions in the gene rather than with methylation. At the same time, in the study of De Martino et al. where cfDNA was analysed by restriction analysis, methylated* VHL* was detected in 50.3% of patients with RCC [19].

Methylation analysis of the* RASSF1*,* FHIT*, and* APC* genes demonstrated their high specificity (93.3% for* RASSF1* and* APC*, 100% for* FHIT*) for renal tumors. Nevertheless, sensitivity in one gene analysis was just from 51.9% for* APC* to 62.9% for* RASSF1* (Table 4). At the same time the use of a combination of three or two genes (without* LRRC3B *due to the low specificity of this gene) leads to a significant increase in sensitivity (77.9–92.3%) and specificity (86.7–93.3%). All other combinations did not reveal any additional diagnosis information. Simultaneous methylation of the* RASSF1*,* APC,* and* FHIT* genes was identified only in 3 patients with metastases. However, the small sample size does not allow us to draw a conclusion on the correlation between methylation and disease progression. At the same time binary logistic regression analysis showed the considerable diagnostic potential of combining both approaches used in this study. According to the ROC analysis the use of only cfDNA concentration has moderate diagnostic potential (AUC = 0.8). On the other hand, by using the concentration and methylation of two or three genes, we achieved 100% diagnostic accuracy in our samples. These results, of course, cannot be directly transferred to clinical practice and need verification on a larger number of samples. However, our data demonstrates the potential advantage there is in combining evaluation of cfDNA concentration and gene methylation for RCC diagnostics and provides a basis for further research.

Thus, despite the small sampling, our results confirm the possibility of using the concentration of cfDNA in blood plasma as an additional marker of renal cancer development and show that methylation of three genes,* FHIT*,* APC*, and* RASSF1*, in cfDNA can be used to develop renal cancer diagnostic tools.

## 5. Conclusion

The results obtained indicate that the concentration of cell-free DNA in plasma and the methylation of specific genes (such as* FHIT*,* APC*, and* RASSF1*) can be a significant addition to serological tumor markers in the identification of patients with renal cancer. However, further studies need to be performed to evaluate their diagnostic value.

## Supplementary Material

Supplementary Figure 1. Control of cfDNA extraction by PCR.
Supplemental Table S1. Experimental details of the qPCR analyses according to the checklist of the MIQE guidelines.

## Figures and Tables

**Table 1:** Patient and tumor characteristics.

	Number of patients
Age at diagnosis:	
Age > 55	20 (74.1%)
Age < 55	7 (25.9%)
Gender:	
Male	13 (48.1%)
Female	14 (51.9%)
Histology:	
Clear cell	23 (85.2%)
Sarcoma-like	2 (7.4%)
Papillary (75%)/clear cell (25%)	1 (3.7%)
Cancer of the renal pelvis	1 (3.7%)
Fuhrman grade:	
G1 + G2	19 (70.4%)
G3 + G4	8 (29.6%)
Clinical stage:	
Stage 2	4 (14.8%)
Stage 3	23 (85.2%)
Tumor-Node-Metastasis (TNM):	
T1a+b N0 M0-X	15 (55.6%)
T2 N0 M0-X	6 (22.2%)
T3 N0-1 M1-X	4 (14.8%)
TNM NA	2 (7.4%)

**Table 2:** Comparative analysis of different methods used to measure cfDNA in blood plasma.

	Method	
	qPCR analysis	SYBR Green I fluorescence measurements
AUC	0.8049 (95% Cl: 0.6602–0.9497)	0.7679 (95% Cl: 0.6242–0.9116)
Median (renal cancer)	80.96	235.5
Median (control)	35.1	53.7
*p* value (by Mann-Whitney *U* test)	*p* < 0.0008	*p* < 0.0037

**Table 3:** Summary of clinicopathological characteristics of patients with RCC and methylation status of *LRRC3B*, *RASSF1*, *FHIT*, and *APC* CpG islands in cfDNA^*∗*^.

Number	Pathology	Age (y)	Sex	pTNM	Clinical grade	Fuhrman nuclear grade	Methylation			
							*LRRC3B*	*RASSF1*	*APC*	*FHIT*
1	ccRCC	54	M	рТ2N0M0	II	3	+	+	−	+
2	ccRCC	61	M	Т1N0M0	II	2	+	+	−	+
3	Sarcoma-like	66	F	рТ2N0MX	II	3	+	−	+	+
4	Papillary/ccRCC	63	M	рТ1вN0MX	III	1	−	+	−	+
5	ccRCC	47	F	рТ3аN0M1	III	3	+	+	+	+
6	ccRCC	64	M	рТ3аN0M1	III	3	+	+	+	+
7	ccRCC	58	M	рТ2N0MX	III	3	+	−	+	−
8	ccRCC	61	M	рТ1вN0M0	III	2	+	+	−	−
9	ccRCC	75	M	рТ1вN0MX	III	2	−	+	−	+
10	ccRСС	65	M	T2N0MX	III	3	−	+	+	+
11	ccRCC	61	F	pT1N0M0	III	2	+	−	+	−
12	ccRCC	63	F	рТ3аN1MX	III	2-3	+	−	+	−
13	ccRCC	68	F	pT1 N0 MX	III	1-2	+	−	+	+
14	ccRCC	34	M	рТ1аN0 MX	III	1	+	−	+	−
15	Cancer of the renal pelvis	76	M	pT3N0M1	III	4	+	+	+	+
16	ccRCC	56	F	рТ1аN0MX	III	1	−	−	+	+
17	ccRCC	62	F	рТ1аN0MX	III	1	+	−	+	+
18	ccRCC	46	F	рТ1вN0MX	III	1	+	+	−	−
19	ccRCC	55	F	рТ2N0MX	III	2	+	+	+	−
20	ccRCC	45	F	T2N0M0	II	2	−	+	−	−
21	ccRCC	61	F	рТ1аN0MX	III	2	+	+	−	−
22	ccRCC	60	M	NA	III	2	+	+	−	+
23	ccRCC	63	F	рТ1вN0MX	III	2	+	−	−	−
24	Sarcoma-like	60	F	NA	III	4	+	−	−	−
25	ccRCC	45	M	pT1вN0MX	III	2	−	+	+	+
26	ccRCC	63	M	рТ1аN0M0	III	1	+	+	−	+
27	ccRCC	73	F	рТ1аN0M0	III	1	−	+	−	−

^*∗*^The results in the Table are presented only for the genes with detected aberrant methylation in cfDNA.

**Table 4:** Diagnostic data analysis for the discrimination of renal cancer patients and healthy subjects using cfDNA methylation of various genes alone and in combination.

Markers	Renal cell carcinoma (*n* = 27)	Healthy controls (*n* = 15)	*χ* ^2^, *p* value	Sensitivity^*∗*^, %	Specificity^*∗∗*^, %
*LRRC3B*	20 (74.1%)	5 (33.3%)	0.01	74.1	66.7
*RASSF1*	17 (63.0 %)	1 (6.7%)	0.0058	62.9	93.3
*FHIT*	15 (55.6%)	0 (0%)	0.0003	55.6	100
*APC*	14 (51.9%)	1 (6.7%)	0.0034	51.9	93.3
*VHL*	0 (0%)	0 (0%)		0	100
*ITGA9*	0 (0%)	0 (0%)		0	100
*RASSF1 *or* FHIT *or* APC*	25 (92.3%)	2 (13.3%)	<0.0001	92.3	86.7
*RASSF1 *or* FHIT*	21 (77.8%)	1 (6.7%)	<0.0001	77.8	93.3
*RASSF1 *or* APC*	21 (77.8%)	1 (6.7%)	<0.0001	77.8	93.3

^*∗*^Sensitivity was calculated as a percentage of positive results from a number of tested RCC patients; ^*∗∗*^specificity was calculated as a percentage of negative tests from a given number of healthy donors.

**Table 5:** Receiver-operating characteristic (ROC) curve analyses of cfDNA marker-based models to discriminate between healthy persons (*n* = 15) and renal cancer patients (*n* = 27)^*∗*^.

	AUC	Std. error	*p* value	95% LCL	95% UCL
Conc_qPCR	0.80494	0.06771	0.00119	0.67223	0.93765
Conc_qPCR+*APC*	0.91852	0.04205	8.61*E* − 06	0.8361	1.00094
Conc_qPCR+*FHIT*	0.91358	0.04898	1.10*E* − 05	0.81759	1.00957
Conc_qPCR+*RASSF1*	0.88148	0.05986	5.00*E* − 05	0.76416	0.99881
Conc_qPCR+ *APC*+*RASSF1*	1	0	1.06*E* − 07	1	1
Conc_qPCR+*APC*+*FHIT*	0.95802	0.03018	1.12*E* − 06	0.89888	1.01717
Conc_qPCR+*RASSF1A*+*FHIT*	0.94568	0.04521	2.16*E* − 06	0.85708	1.03428
Conc_qPCR+*APC*+*FHIT*+*RASSF1*	1	0	1.06*E* − 07	1	1

^*∗*^Calculated by binary logistic regression using combination of different markers: concentration of cfDNA determined by qPCR (Conc_qPCR) and methylation marker genes (*APC*, *FHIT*, and *RASSF1*).

## References

[1] Banumathy G., Cairns P. (2010). Signaling pathways in renal cell carcinoma. *Cancer Biology and Therapy*.

[2] Gupta K., Miller J. D., Li J. Z., Russell M. W., Charbonneau C. (2008). Epidemiologic and socioeconomic burden of metastatic renal cell carcinoma (mRCC): a literature review. *Cancer Treatment Reviews*.

[3] Ministry of Healthcare of Ukraine (2011). *The Features Diagnosis and Prognosis Factors Renal Cell Carcinoma*.

[4] World Cancer Research Fund International/American Institute for Cancer Research (2015). *Continuous Update Project Report: Diet, Nutrition, Physical Activity and Kidney Cancer*.

[5] Lopez-Beltran A., Scarpelli M., Montironi R., Kirkali Z. (2006). 2004 WHO classification of the renal tumors of the adults. *European Urology*.

[6] Hadaczek P., Siprashvili Z., Markiewski M. (1998). Absence or reduction of FHIT expression in most clear cell renal carcinomas. *Cancer Research*.

[7] Arai E., Kanai Y. (2011). Genetic and epigenetic alterations during renal carcinogenesis. *International Journal of Clinical and Experimental Pathology*.

[8] Lasseigne B. N., Burwell T. C., Patil M. A., Absher D. M., Brooks J. D., Myers R. M. (2014). DNA methylation profiling reveals novel diagnostic biomarkers in renal cell carcinoma. *BMC Medicine*.

[9] Shenoy N., Vallumsetla N., Zou Y. (2015). Role of DNA methylation in renal cell carcinoma. *Journal of Hematology and Oncology*.

[10] Fournié G. J., Courtin J.-P., Laval F. (1995). Plasma DNA as a marker of cancerous cell death. Investigations in patients suffering from lung cancer and in nude mice bearing human tumours. *Cancer Letters*.

[11] Schwarzenbach H., Hoon D. S. B., Pantel K. (2011). Cell-free nucleic acids as biomarkers in cancer patients. *Nature Reviews Cancer*.

[12] Taback B., Hoon D. S. B. (2004). Circulating nucleic acids and proteomics of plasma/serum: clinical utility. *Annals of the New York Academy of Sciences*.

[13] Wasserkort R., Kalmar A., Valcz G. (2013). Aberrant septin 9 DNA methylation in colorectal cancer is restricted to a single CpG island. *BMC Cancer*.

[14] Skrypkina I. Ya., Kashuba V. I., Gordiyuk V. V. (2006). Identification of changes in gene loci potentially associated with renal cancer by novel technique of NotI microarrays. *Reports of the National Academy of Sciences of Ukraine*.

[15] Kashuba V. I., Skrypkina I. Y., Saraev D. V. (2006). Identification of changes in gene loci potentially associated with cervical cancer using NotI microarrays. *Ukrain'skyi Biokhimichnyi Zhurnal*.

[16] Loginov V. I., Khodyrev D. S., Pronina I. V. (2009). Methylation of the RASSF1A promoter region and the allelic imbalance frequencies in chromosome 3 critical regions correlate with progression of clear cell renal carcinoma. *Molecular Biology*.

[17] Peters I., Rehmet K., Wilke N. (2007). RASSF1A promoter methylation and expression analysis in normal and neoplastic kidney indicates a role in early tumorigenesis. *Molecular Cancer*.

[18] Hauser S., Zahalka T., Fechner G. (2013). Serum DNA hypermethylation in patients with kidney cancer: results of a prospective study. *Anticancer Research*.

[19] De Martino M., Klatte T., Haitel A., Marberger M. (2012). Serum cell-free DNA in renal cell carcinoma: a diagnostic and prognostic marker. *Cancer*.

[20] Hoque M. O., Begum S., Topaloglu O. (2004). Quantitative detection of promoter hypermethylation of multiple genes in the tumor, urine, and serum DNA of patients with renal cancer. *Cancer Research*.

[21] Ramp U., Caliskan E., Ebert T. (2002). FHIT expression in clear cell renal carcinomas: versatility of protein levels and correlation with survival. *The Journal of Pathology*.

[22] Kvasha S., Gordiyuk V., Kondratov A. (2008). Hypermethylation of the 5′CpG island of the *FHIT* gene in clear cell renal carcinomas. *Cancer Letters*.

[23] Herrera L. J., Raja S., Gooding W. E. (2005). Quantitative analysis of circulating plasma DNA as a tumor marker in thoracic malignancies. *Clinical Chemistry*.

[24] Czeiger D., Shaked G., Eini H. (2011). Measurement of circulating cell-free DNA levels by a new simple fluorescent test in patients with primary colorectal cancer. *American Journal of Clinical Pathology*.

[25] Herman J. G., Graff J. R., Myöhänen S., Nelkin B. D., Baylin S. B. (1996). Methylation-specific PCR: a novel PCR assay for methylation status of CpG islands. *Proceedings of the National Academy of Sciences of the United States of America*.

[26] Lo K., Kwong J., Hui A. B. (2001). Advances in brief high frequency of promoter hypermethylation of RASSF1A in nasopharyngeal carcinoma. *Journal of Clinical Oncology*.

[27] Zöchbauer-Müller S., Fong K. M., Maitra A. (2001). 5′ CpG island methylation of the FHIT gene is correlated with loss of gene expression in lung and breast cancer. *Cancer Research*.

[28] Shinozaki M., Hoon D. S. B., Giuliano A. E. (2005). Distinct hypermethylation profile of primary breast cancer is associated with sentinel lymph node metastasis. *Clinical Cancer Research*.

[29] Kondratov A. G., Stoliar L. A., Kvasha S. M. (2012). Methylation pattern of the putative tumor-suppressor gene LRRC3B promoter in clear cell renal cell carcinomas. *Molecular Medicine Reports*.

[30] Kuroki T., Trapasso F., Yendamuri S. (2003). Allele loss and promoter hypermethylation of VHL, RAR-*β*, RASSF1A, and FHIT tumor suppressor genes on chromosome 3p in esophageal squamous cell carcinoma. *Cancer Research*.

[31] Li J.-L., Fei Q., Yu J., Zhang H.-Y., Wang P., De Zhu J. (2004). Correlation between methylation profile of promoter cpg islands of seven metastasis-associated genes and their expression states in six cell lines of liver origin. *Ai Zheng*.

[32] Bhalerao S., Kadam P. (2010). Sample size calculation. *International Journal of Ayurveda Research*.

[33] Heitzer E., Ulz P., Geigl J. B. (2015). Circulating tumor DNA as a liquid biopsy for cancer. *Clinical Chemistry*.

[34] Roth C., Pantel K., Müller V. (2011). Apoptosis-related deregulation of proteolytic activities and high serum levels of circulating nucleosomes and DNA in blood correlate with breast cancer progression. *BMC Cancer*.

[35] Sai S., Ichikawa D., Tomita H. (2007). Quantification of plasma cell-free DNA in patients with gastric cancer. *Anticancer Research*.

[36] Wu T.-L., Zhang D., Chia J.-H., Tsao K.-C., Sun C.-F., Wu J. T. (2002). Cell-free DNA: measurement in various carcinomas and establishment of normal reference range. *Clinica Chimica Acta*.

[37] Bettegowda C., Sausen M., Leary R. J. (2014). Detection of circulating tumor DNA in early- and late-stage human malignancies. *Science Translational Medicine*.

[38] Feng G., Ye X., Fang F., Pu C., Huang H., Li G. (2013). Quantification of plasma cell-free DNA in predicting therapeutic efficacy of sorafenib on metastatic clear cell renal cell carcinoma. *Disease Markers*.

[39] Kienel A., Porres D., Heidenreich A., Pfister D. (2015). cfDNA as a prognostic marker of response to taxane based chemotherapy in patients with prostate cancer. *The Journal of Urology*.

[40] Chen Z., Fadiel A., Naftolin F., Eichenbaum K. D., Xia Y. (2005). Circulation DNA: biological implications for cancer metastasis and immunology. *Medical Hypotheses*.

[41] Jung K., Fleischhacker M., Rabien A. (2010). Cell-free DNA in the blood as a solid tumor biomarker—a critical appraisal of the literature. *Clinica Chimica Acta*.

[42] Kamat A. A., Bischoff F. Z., Dang D. (2006). Circulating cell-free DNA: a novel biomarker for response to therapy in ovarian carcinoma. *Cancer Biology and Therapy*.

[43] Cheng C., Omura-Minamisawa M., Kang Y., Hara T., Koike I., Inoue T. (2009). Quantification of circulating cell-free DNA in the plasma of cancer patients during radiation therapy. *Cancer Science*.

[44] Thierry A. R., Mouliere F., Gongora C. (2010). Origin and quantification of circulating DNA in mice with human colorectal cancer xenografts. *Nucleic Acids Research*.

[45] Mouliere F., Robert B., Peyrotte E. (2011). High fragmentation characterizes tumour-derived circulating DNA. *PLoS ONE*.

[46] Pinzani P., Salvianti F., Zaccara S. (2011). Circulating cell-free DNA in plasma of melanoma patients: qualitative and quantitative considerations. *Clinica Chimica Acta*.

[47] Lu H., Busch J., Jung M. (2016). Diagnostic and prognostic potential of circulating cell-free genomic and mitochondrial DNA fragments in clear cell renal cell carcinoma patients. *Clinica Chimica Acta*.

[48] Hauser S., Zahalka T., Ellinger J. (2010). Cell-free circulating DNA: diagnostic value in patients with renal cell cancer. *Anticancer Research*.

[49] Wan J., Zhu L., Jiang Z., Cheng K. (2013). Monitoring of plasma cell-free DNA in predicting postoperative recurrence of clear cell renal cell carcinoma. *Urologia Internationalis*.

[50] Fleischhacker M., Schmidt B. (2007). Circulating nucleic acids (CNAs) and cancer—a survey. *Biochimica et Biophysica Acta (BBA)—Reviews on Cancer*.

[51] Levenson V. V. (2010). DNA methylation as a universal biomarker. *Expert Review of Molecular Diagnostics*.

[52] Dworkin A. M., Huang T. H.-M., Toland A. E. (2009). Epigenetic alterations in the breast: implications for breast cancer detection, prognosis and treatment. *Seminars in Cancer Biology*.

[53] Hoque M. O. (2009). DNA methylation changes in prostate cancer: current developments and future clinical implementation. *Expert Review of Molecular Diagnostics*.

[54] Warton K., Samimi G. (2015). Methylation of cell-free circulating DNA in the diagnosis of cancer. *Frontiers in Molecular Biosciences*.

[55] Anglim P. P., Alonzo T. A., Laird-Offringa I. A. (2008). DNA methylation-based biomarkers for early detection of non-small cell lung cancer: an update. *Molecular Cancer*.

[56] Delpu Y., Cordelier P., Cho W. C., Torrisani J. (2013). DNA methylation and cancer diagnosis. *International Journal of Molecular Sciences*.

[57] Charlton J., Williams R. D., Weeks M. (2014). Methylome analysis identifies a Wilms tumor epigenetic biomarker detectable in blood. *Genome Biology*.

[58] Brown T. C., Juhlin C. C., Healy J. M., Prasad M. L., Korah R., Carling T. (2014). Frequent silencing of RASSF1A via promoter methylation in follicular thyroid hyperplasia: a potential early epigenetic susceptibility event in thyroid carcinogenesis. *JAMA Surgery*.

[59] Joo M. K., Kim K. H., Park J.-J. (2015). CpG island promoter hypermethylation of Ras association domain family 1A gene contributes to gastric carcinogenesis. *Molecular Medicine Reports*.

[60] Wu X.-M., Chen Y., Shao Y., Zhou X.-L., Tang W.-R. (2014). Association between cigarette smoking and RASSF1A gene promoter hypermethylation in lung cancer patients: a meta- analysis. *Asian Pacific Journal of Cancer Prevention*.

[61] Wang H.-L., Liu P., Zhou P.-Y., Zhang Y. (2014). Promoter methylation of the RASSF1A gene may contribute to colorectal cancer susceptibility: a meta-analysis of cohort studies. *Annals of Human Genetics*.

[62] Si J.-G., Su Y.-Y., Han Y.-H., Chen R.-H. (2014). Role of RASSF1A promoter methylation in the pathogenesis of ovarian cancer: a meta-analysis. *Genetic Testing and Molecular Biomarkers*.

[63] Daniunaite K., Jarmalaite S., Kalinauskaite N. (2014). Prognostic value of RASSF1 promoter methylation in prostate cancer. *The Journal of Urology*.

[64] Shi H., Li Y., Wang X. (2013). Association between RASSF1A promoter methylation and ovarian cancer: a meta-analysis. *PLoS ONE*.

[65] Spugnardi M., Tommasi S., Dammann R., Pfeifer G. P., Hoon D. S. B. (2003). Epigenetic inactivation of RAS association domain family protein 1 (RASSF1A) in malignant cutaneous melanoma. *Cancer Research*.

[66] Ellinger J., Holl D., Nuhn P. (2011). DNA hypermethylation in papillary renal cell carcinoma. *BJU International*.

[67] Gordiyuk V. V., Gerashchenko G. V., Skrypkina I. Y. (2008). Identification of chromosome 3 epigenetic and genetic abnormalities and gene expression changes in ovarian cancer. *Biopolymers and Cell*.

[68] Gerashchenko G. V., Gordiyuk V. V., Skrypkina I. Y. (2009). Screening of epigenetic and genetic disturbances of human chromosome 3 genes in colorectal cancer. *Ukrainskiĭ Biokhimicheskiĭ Zhurnal*.

[69] Kim M., Kim J.-H., Jang H.-R. (2008). LRRC3B, encoding a leucine-rich repeat-containing protein, is a putative tumor suppressor gene in gastric cancer. *Cancer Research*.

[70] Kondratov A. G., Nekrasov K. A., Lototska L. V. (2014). Comparative analysis of epigenetic markers in plasma and tissue of patients with colorectal cancer. *Biopolymers and Cell*.

[71] Demokan S., Chuang A. Y., Pattani K. M., Sidransky D., Koch W., Califano J. A. (2014). Validation of nucleolar protein 4 as a novel methylated tumor suppressor gene in head and neck cancer. *Oncology Reports*.

[72] Takada Y., Ye X., Simon S. (2007). The integrins. *Genome Biology*.

[73] Mambole A., Bigot S., Baruch D., Lesavre P., Halbwachs-Mecarelli L. (2010). Human neutrophil integrin *α*9*β*1: up-regulation by cell activation and synergy with *β*2 integrins during adhesion to endothelium under flow. *Journal of Leukocyte Biology*.

[74] Stupack D. G., Cheresh D. A. (2004). Integrins and angiogenesis. *Current Topics in Developmental Biology*.

[75] Rathinam R., Alahari S. K. (2010). Important role of integrins in the cancer biology. *Cancer and Metastasis Reviews*.

[76] Ghosh A., Ghosh S., Maiti G. P. (2010). Frequent alterations of the candidate genes hMLH1, ITGA9 and RBSP3 in early dysplastic lesions of head and neck: clinical and prognostic significance. *Cancer Science*.

[77] Häkkinen L., Kainulainen T., Salo T., Grenman R., Larjava H. (1999). Expression of integrin *α*9 subunit and tenascin in oral leukoplakia, lichen planus, and squamous cell carcinoma. *Oral Diseases*.

[78] Roy S., Bingle L., Marshall J. F. (2011). The role of *α*9*β*1 integrin in modulating epithelial cell behaviour. *Journal of Oral Pathology and Medicine*.

[79] Høye A. M., Couchman J. R., Wewer U. M., Fukami K., Yoneda A. (2012). The newcomer in the integrin family: integrin *α*9 in biology and cancer. *Advances in Biological Regulation*.

[80] Mitra S., Indra D. M., Bhattacharya N. (2010). *RBSP3* is frequently altered in premalignant cervical lesions: clinical and prognostic significance. *Genes Chromosomes and Cancer*.

