# Supplementary material for: Concentration and Methylation of Cell-Free DNA from Blood Plasma as Diagnostic Markers of Renal Cancer
Source: Dis Markers. 2016 Sep 20;2016:3693096. doi: 10.1155/2016/3693096 (PMC5048037; doi:10.1155/2016/3693096)
Supplement: Supplementary file 1 — Supplementary Figure 1. Control of cfDNA extraction by PCR. Supplemental Table S1. Experimental details of the qPCR analyses according to the checklist of the MIQE guidelines. [file 3693096.f1.pdf]

Supplemental Table S1. Experimental details of the qPCR analyses according to the checklist of the MIQE* guidelines.

ITEM TO CHECK	IMPOR-TANCE	CHECK-LIST	WHERE IN THE MANUSCRIPT; ADDITIONAL COMMENT
**ERXPERIMENTAL**			
Definition of experimental and control groups	E	Yes	2. Materials and Methods: 2.1. Sample Collection. 3. Results: Table 1, 3
Number within each group	E	Yes	2. Materials and methods: 2.1. Sample Collection
Assay carried out by core lab or investigator´s lab?	D	Yes	All assays were performed in investigator´s lab.
Acknowledgement of authors´ contributions	D	Yes	see Acknowledgements.
**SAMPLE**			
Description	E	Yes	2. Materials and Methods: 2.1. Sample Collection
Volume/mass of sample processed	D	Yes	2. Materials and Methods: 2.2. Extraction of cfDNA , 5 ml blood
Microdissection or macrodissection	E	N/A	
Processing procedure	E	Yes	2. Materials and Methods: 2.2. Extraction of cfDNA.
If frozen – how and how quickly?	E	Yes	2. Materials and Methods: 2.2. Extraction of cfDNA.
If fixed – with what, how quickly?	E	N/A	
Samples storage conditions and duration (esp. for FFPE samples)	E	Yes	2. Materials and Methods: 2.2. Extraction of cfDNA.
**NUCLEIC ACID EXTRACTION**			
Procedure and/or instrumentation	E	Yes	2. Materials and Methods: 2.2. Extraction of cfDNA.
Name of kit and details of any modifications	E	Yes	2. Materials and Methods: 2.2. Extraction of cfDNA.
Source of additional reagents used	E	Yes	No additional reagents.
Details of DNase or RNase treatment	E	Yes	no RNase treatment
Contamination assessment (DNA or RNA)	E	Yes	Materials and Methods.
Nucleic acid quantification	E	Yes	See Supplemenatry Figure S1: Control extraction of cfDNA by PCR
Instrument and method	E	Yes	2. Materials and Methods: 2.2. Extraction of cfDNA.
Purity (A260/A280)	D	Yes	Low concentrations cfDNA did not allow reliable absorbance measurements.
Yield	D	No	
RNA integrity method/instrument	E	N/A	
RIN/RQI or Cq of 3`and 5`transcripts	E	N/A	
Electrophoresis traces	D	N/A	
Inhibition testing (Cq dilutions, spike or other)	E	Yes	Dilution experiments were performed; PCR efficiencies were found 100. High efficiencies of PCR was obtained for diluted cfDNA (1:5).
**REVERSE TRANSCRIPTION**			
Complete reaction condition	E	N/A	
Amount of RNA and reaction volume	E	N/A	
Priming oligonucleotide (if using GSP) and concentration	E	N/A	
Reverse transcriptase and concentration	E	N/A	

ITEM TO CHECK	IMPOR-TANCE	CHECK-LIST	WHERE IN THE MANUSCRIPT; ADDITIONAL COMMENT
Temperature and time	E	N/A	
Manufacturer and reagents and catalogue numbers	D	N/A	
Cqs with and without RT	D*	N/A	
Storage conditions of cDNA	D	N/A	
**qPCR TARGET INFORMATION**			
If multiplex, efficiency and LOD of each assay	E	N/A	
Sequence accession number	E	Yes	Homo sapiens actin beta (*ACTB*), chr. 7p22, GeneID:60, accession NC_000007, NM_001101.
Location of amplicon	D	Yes	2. Materials and Methods: 2.3. Quantification of plasma cfDNA by real-time PCR. See Herrera et al. [ref.23 in the text]. 5528505-5528603 bp on NC_000007.14 Homo sapiens chromosome 7, GRCh38.p7 Primary Assembly
Amplicon length	E	Yes	99 bp
In silico specificity screen (BLAST, etc.)	E	Yes	See Herrera et al. [ref.23 in the text]. See Supplemenatry Figure S1: Control extraction of cfDNA by PCR
Pseudogenes, retropseudogenes or other homologs?	D	Yes	2. Materials and Methods: 2.3. Quantification of plasma cfDNA by real-time PCR. See Herrera et al. [ref.23 in the text]. Use of cfDNA specific TaqMan assays
Sequence alignment	D	Yes	See Herrera et al. [ref.23 in the text]
Secondary structure analysis of amplicon	D	N/A	
Location of each primer by exon or intron (if applicable)	E	Yes	The primers location by exon 4 of beta-actin (*ACTB*)mRNA, NM_001101
What splice variants are targeted?	E	Yes	Primers were designed to amplify genomic region of *ACTB* gene
**qPCR OLIGONUCLEOTIDES**			
Primer sequences	E	Yes	2. Materials and Methods: 2.3. Quantification of plasma cfDNA by real-time PCR.
RTPrimerDB Identification Number	D	No	
Probe sequences	D**	Yes	2. Materials and Methods: 2.3. Quantification of plasma cfDNA by real-time PCR.
Location and identity of any modifications	E	Yes	2. Materials and Methods: 2.3. Quantification of plasma cfDNA by real-time PCR. See Herrera et al. [ref.23 in the text].
Manufacture of oligonucleotides	D	Yes	Invitrogen Life Technologies, USA
Purification method	D	Yes	HPLC

ITEM TO CHECK	IMPOR-TANCE	CHECK-LIST	WHERE IN THE MANUSCRIPT; ADDITIONAL COMMENT
**qPCR PROTOCOL**			
Complete reaction conditions	E	Yes	qPCRs were performed in a BioRad iQ5 Real-Time PCR System (Bio-Rad, USA) using 0.2 ml PCR tubes Axygen Scientific, USA (Cat. No. PCR-02D-C). The qPCR reaction mix (V=20 µl) consisted of sterile H_2O MilliQ, 0.3 µM of each primer, 0.25 µM TaqMan probe, 3,0 mM Mg^{2+}, 0.3 mM dNTPs,, 1X Hot Start PCR buffer and 0.06 U Maxima Hot Start Taq DNA Polymerase (Thermo Scientific, USA) and 5 µl of 1:5–diluted cfDNA/standard DNA. The qPCR amplification was performed starting with an initial activation step (95°C for 10 min) followed by 40 cycles of 95°C for 15 s and 60°C for 60 s
Reaction volume and amount of cDNA/DNA	E	Yes	20 µl. Amount of cfDNA or standart DNA: 5 µl of 1:5–diluted in sterile H_2O MilliQ
Primer, (probe), Mg++ and dNTP concentration	E	Yes	0.3 µM of each primer, 0.25 µM Tm probe, 3,0 mM Mg^{2+} (25 mM $MgCl_2$ supplied to Maxima Hot Start Taq DNA Polymerase, Cat.No. EP0601, Thermo Scientific, USA), 0.3 mM dNTPs (dNTP Mix (10 mM each), Cat.No. R0191, Thermo Scientific, USA.). See Herrera et al. [ref.23 in the text].
Polymerase identity and concentration	E	Yes	Maxima Hot Start Taq DNA Polymerase (Cat.No. EP0601, Thermo Scientific, USA) 0.06 units per reaction
Buffer/kit identity and manufacture	E	No	1X Hot Start PCR buffer (supplied to Maxima Hot Start Taq DNA Polymerase, Cat.No. EP0601,Thermo Scientific, USA)
Exact chemical constitution of the buffer	D	Yes	200 mM Tris HCl (pH 8.3 at 25°C), 200 mM KCl, 50 mM (NH4)2SO4 (supplied to Cat.No. EP0601,Thermo Scientific, USA)
Additives (SYBR Green I, DMSO, etc.)	E	Yes	No additives
Manufacturer of plates/tubes and catalog number	D	Yes	0.2 ml PCR tubes of Axygen Scientific, USA (Cat. No. PCR-02D-C) with sealing foils)
Complete thermocycling parameter	E	Yes	10 min at 95°C, followed by 40 cycles of 15 s at 95°C and 1 min at 60°C.
Reaction setup (manual/robotic)	D	Yes	Manual setup.
Manufacturer of qPCR instruments	E	Yes	BioRad iQ5 Real-Time PCR System (BioRad, USA)
**qPCR VALIDATION**			
Evidence of optimisation (from gradients)	D	Yes	See Herrera et al. [ref.23 in the text].
Specificity (gel, sequence, melt, or digest)	E	Yes	Agarose gel analysis (Supplementary Fig. S1) and sequence<br>Using of TaqMan probe enhanced specificity of PCR
For SYBR Green I, Cq of the NTC	E	N/A	
Calibration curves with slope and Y-intercept	E	Yes	Slope= - 3.327 and Y-intercept=34.025
PCR efficiency calculated from slope	E	Yes	E=99,8%
Confidence interval PCR efficiency or standard error	D	Yes	2. Material and Methods: 2.3. Quantification of plasma cfDNA by real-time PCR

ITEM TO CHECK	IMPOR-TANCE	CHECK-LIST	WHERE IN THE MANUSCRIPT; ADDITIONAL COMMENT
r2 of standard curve	E	Yes	2. Material and Methods: 2.3. Quantification of plasma cfDNA by real-time PCR
Linear dynamic range	E	Yes	2. Material and Methods: 2.3. Quantification of plasma cfDNA by real-time PCR
Cq variation at lower limit	E	Yes	Not determined since Cqs of the samples were in the dynamic range below the lowest concentration.
Confidence intervals throughout range	D	Yes	Not characterized
Evidence for limit of detection	E	Yes	Not determined since Cqs of the samples were in the dynamic range below the lowest concentration. Cq dynamic range between the highest and lowest values for Cq of the samples was 28,07- 37,53
If multiplex, efficiency and LOD of each assay	E	N/A	
**DATA ANALYSIS**			
qPCR analysis program (source, version)	E	Yes	Bio-Rad iQ5™ Optical System Software Version 2.0 (Bio-Rad, USA)
Cq method determination	E	Yes	Cq was calculated by determining the threshold. The same threshold was taken for all samples
Outlier identification and disposition	E	Yes	2. Material and Methods: 2.3. Quantification of plasma cfDNA by real-time PCR
Results of NTCs	E	Yes	NTC did not result in any amplification; Cq >40.
Justification of number and choice of reference genes	E	N/A	
Description of normalization method	E	No	2. Material and Methods: 2.6. Statistical analysis
Number and concordance of biological replicates	D		2. Materials and Methods: 2.1. Sample Collection. 3. Results: Table 1, 3
Number and stage (RT or qPCR) of technical replicates	E	Yes	2. Material and Methods: 2.3. Quantification of plasma cfDNA by real-time PCR.
Repeatability (intra-assay variation, %CV)	E	Yes	Assay variations (n=8) was based on CP variation: CPmean was 22.76, CPstandard deviation ±0.17, %CV - 0.7
Reproducibility (inter-assay variation, %CV)	D	No	
Power analysis	D	No	
Statistical methods for result significance	E	Yes	2. Materials and Methods: 2.6. Statistical analysis
Software (source, version)	E	Yes	Bio-Rad iQ™5 Optical System Software, Version 2.1.148.060623. 2. Materials and Methods: 2.6. Statistical analysis.
Cq or raw data submission RDML	D	No	

*The checklist of the characteristics of the qPCR analyses according to Bustin et al. [1].

E: Essential information, D: Desirable information, N/A: Not applicable

[1] Bustin SA, Benes V, Garson JA, Hellemans J, Huggett J, Kubista M, et al. The MIQE guidelines: minimum information for publication of quantitative real-time PCR experiments. Clin Chem 2009;55:611-22

Supplementary Figure S1. Control of cfDNA extraction by PCR

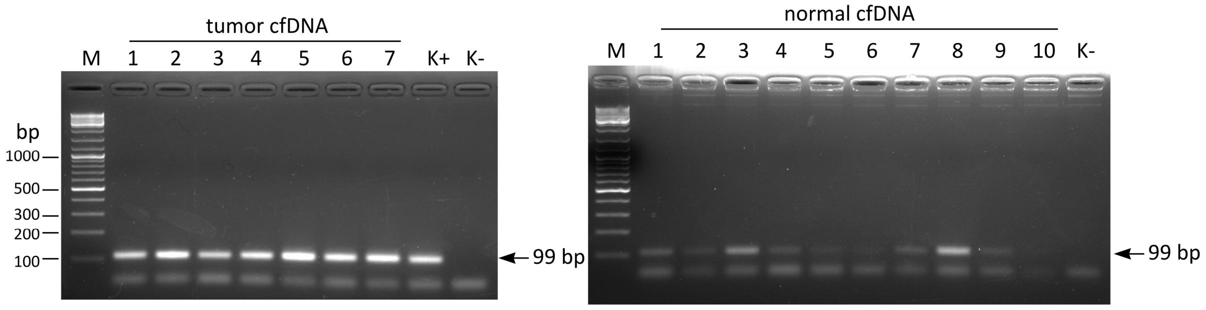

PCR was performed in Applied Biosystems 2720® thermal cycler *(*Applied Biosystems*,* USA). PCR products were analyzed by ethidium bromide stained agarose gels electrophoresis using Wide Mini-Sub Cell GT Cell and PowerPac Basic Power Supply, 100–120/220–240 V (Bio-Rad, USA). Detection of the gel images was carried out at ChemiDoc™ XRS+ System (Bio-Rad, USA). Primers for 99 bp fragment of *ACTB* gene: 5′-CCACACTGTGCCCATCTACG-3′ and 5′-AGGATCTTCATGAGGTAGTCAGTCAG-3′.

2% agarose gel electrophoresis: Agarose Low EEO, Cat.No. CSL-AG500, Cleaver Scientific Ltd, UK. Tumor cfDNA, cfDNA blood plasma of kidney cancer patients; normal cfDNA,cfDNA blood plasma of no tumor donors; M, DNA-Molecular Weight Marker "GeneRuler™ DNA Ladder Mix" (Cat.No. SM0331, Thermo Scientific, USA). The ladder is supplied with 6X DNA Loading Dye.
